# Swinging beats: transient heart block in cardiac lymphoma

**DOI:** 10.1007/s12471-018-1135-z

**Published:** 2018-07-23

**Authors:** J. W. Buikema, W. R. Goodyer, S. Koudstaal, J. van ’t Sant, P. W. Verheggen, E. A. de Vrey, B. J. de Smet

**Affiliations:** 1 Department of Cardiology, Meander Medisch Centrum, Amersfoort, The Netherlands; 2 Department of Cardiology, University Medical Center Utrecht, Utrecht, The Netherlands; 3 Stanford Cardiovascular Institute, Stanford University School of Medicine, Stanford, CA USA

**Fig. 1:**
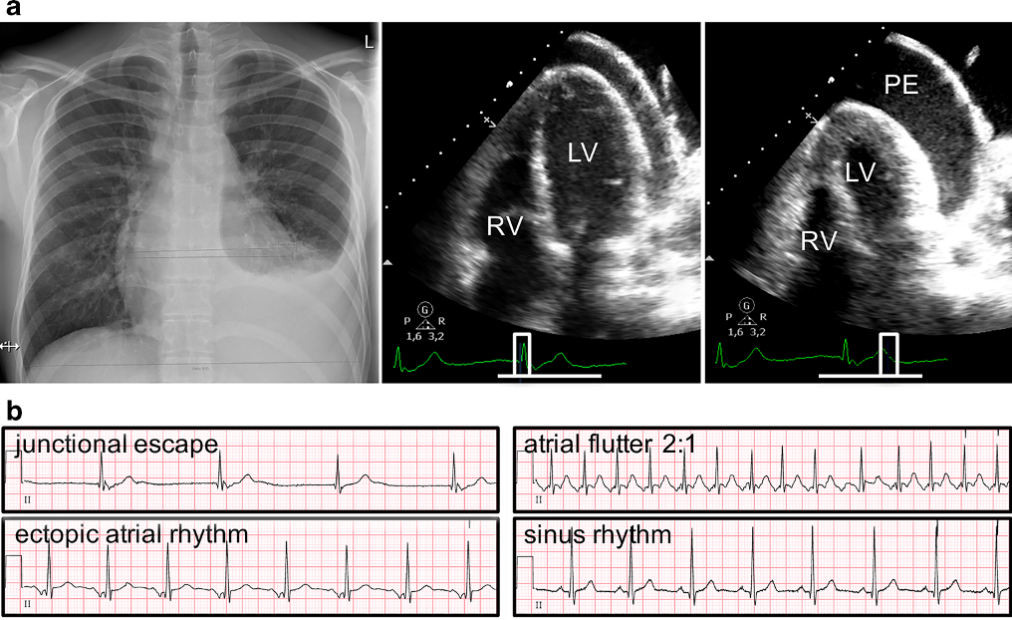
**a** *Left* X‑ray showing enlarged cardiac silhouette and predominantly left-sided pleural effusion. *Middle and right* Transthoracic echocardiography demonstrating large circumferential pericardial effusion with a so-called swinging heart with inflow obstruction. *RV* Right ventricle, *LV* left ventricle, *PE* pleural effusion. **b** Follow-up electrocardiograms showing various ectopic (atrial) foci rhythms before returning to sinus rhythm 6 months later

A 20-year-old man receiving chemotherapy for diffuse large B‑cell lymphoma with vascular involvement presented to the emergency room with dyspnoea. Chest radiography showed left-sided pleural effusion and an enlarged cardiac silhouette. Transthoracic echocardiography demonstrated large circumferential pericardial effusion with a so-called swinging heart with inflow obstruction (Fig. 1a). Remarkably, the patient was bradycardic and follow-up electrocardiography (ECG) revealed a junctional escape rhythm. Symptoms resolved after pericardiocentesis and drainage of 1,100 ml pericardial fluid containing B cells. Despite the drainage, a junctional rhythm persisted and after 24 h of continuous ECG the patient was discharged. Several weeks later, drainage was repeated for recurring pericardial effusion. ECG then showed an atrial flutter with 2:1 conduction. While in remission, follow-up electrocardiograms showed various ectopic atrial foci rhythms before returning to sinus rhythm 6 months later (Fig. 1b). Cardiac involvement of lymphomas is not uncommon. However, when patients develop transient blocks or arrhythmias this can be life-threatening and require additional vigilance during management [1–3].
